# Interpersonal trust: an event-based account

**DOI:** 10.3389/fpsyg.2015.01399

**Published:** 2015-09-15

**Authors:** Bernhard Hommel, Lorenza S. Colzato

**Affiliations:** Cognitive Psychology Unit and Leiden Institute for Brain and Cognition, Leiden UniversityLeiden, Netherlands

**Keywords:** trust, trust game, social behavior, theory, interpersonal relations

Trusting other people is essential for modern societies, in which the sheer complexity of interpersonal relationships renders more traditional control-based strategies of interpersonal cooperation increasingly inefficient (Luhmann, 1979). There is no agreed-upon standard definition of the concept of trust, but the key idea is that “trusting a person means believing that when offered the chance, he or she is not likely to behave in a way that is damaging to us” (Gambetta, 1988, p. 219). While beliefs need not necessarily be supported by reasons, people often do trust a trustee more in the face of information that allows predicting his or her behavior. This means that a core aspect of trust consists in social predictability.

How does trust work? In the following, we suggest that trust reflects the absence of aversive uncertainty, which in turn depends on the degree to which the representation of another person overlaps with a representation of oneself. Based on the theory of event coding (Hommel et al., 2001) we explain how people represent themselves and others, how representational overlap determines trust, how that is affected by the situational context and the trustor's current mindset, and what this implies for interventions to induce and increase interpersonal trust.

## Representing oneself and others

The Theory of Event Coding (TEC: Hommel et al., 2001; Hommel, 2009) aims to explain how people represent events they perceive and events they produce—perceptions and actions that is. In a nutshell, TEC claims that: (a) Perceptual events and planned actions are cognitively represented by *event codes*; (b) which are integrated assemblies of *feature codes* (Hommel, 2004); (c) which in turn are cognitive/brain states correlated with external (perceived or self-generated) features (*distal coding*: Prinz, 1992); (d) which implies that the basic units of both perception and action (assemblies of feature codes) are *sensorimotor* entities, in the sense that they are activated by sensory input (= perception) and controlling motor output (= action).

**Figure 1:**
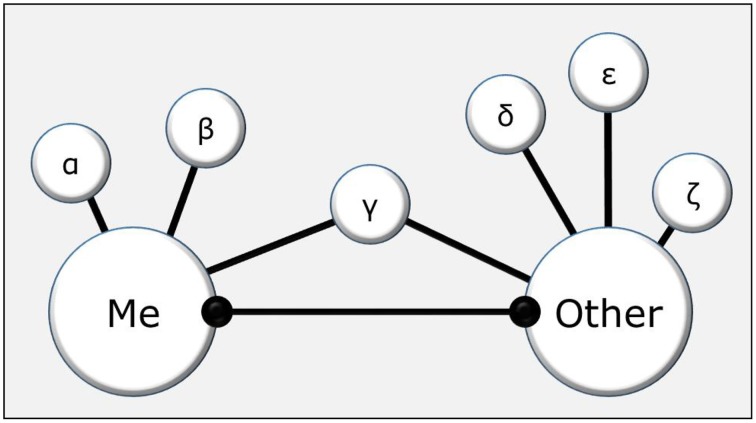
**Representation of oneself (Me) and another person (Other) in terms of situationally relevant features, of which one (γ) is shared**. The relationship between the two representations is determined by (a) the structural feature overlap (the number of all features that are shared); (b) the situationally primed (“intentionally weighted”) features; and (c) the inclusivity/exclusivity of the processing mode.

Originally, TEC has been developed to represent relatively simple stimuli and actions but more recently we have begun to explore whether and how the theory can be extended to represent social events. Interestingly, Greenwald et al. (2002) have suggested a theoretical account of self-representation that seems perfectly compatible with such a social extension of TEC. It is true that representing people and social events would necessarily require a greater complexity of the representational dimensions than was necessary for TECs original focus on colors, shapes, and other simple features, but TECs basic architecture and computational principles allow for that. In particular, TEC provides the mechanisms needed to represent oneself in terms of one's perceptual and action-contingent features and the same is true for other individuals (Hommel et al., 2009; Hommel, 2013). The basic idea is sketched in Figure 1: In the example, an agent is assumed to represent herself (Me node) with respect to 3 features (α, β, γ—e.g., being female, tall, and a student) and another person (Other) with respect to 4 features (γ, δ, ε, ζ—e.g., being a student, male, short, and a father).

The relationship between representations of one person (including oneself) and another is determined by three factors. First, two representations can structurally overlap to different degrees, depending on the number of features they share. In the example, only one feature is shared, which creates only little overlap. If the agent would meet another tall, female student, the overlap will be considerable. Second, features that relate to the agent's current intentions are “primed” (i.e., receive higher “intentional weighting,” cf., Memelink and Hommel, 2013), which enables or increases their impact on cognitive processes. It is because of this mechanism that only a few features are considered in the example. In addition to the three mentioned features, the agent will have many more features, such as a particular hair color, hairdo, age, skin color, dress, and so forth. The example assumes that all these additional features are currently not important (and not particularly salient, which would attract attention exogenously) and, therefore, effectively “invisible.” Third, people have cognitive control over their style of decision-making by biasing it to be either more “exclusive” or more “inclusive” (Colzato et al., 2013b; Hommel, 2015). Technically, this can be expressed as the mutual inhibition between alternative events (indicated by the symmetric inhibitory link between Me and Other), which can be either strong (as in the exclusive mode) or weak (as in the inclusive mode). An inclusive mode would thus tend to treat Me and Other as one event (similar to visual grouping according to Gestalt principles) while an exclusive mode would tend to render them separate events (Hommel, 2015).

## Predictability and trust

Our main hypothesis is that the degree of interpersonal similarity (i.e., the functional overlap between Me and Other) determines the degree of trust for the Other. The starting point of our consideration is the assumption that trust depends on predictability by definition—the better we can predict the behavior of someone, the better we can judge whether “he or she is not likely to behave in a way that is damaging to us” (Gambetta, 1988, p. 219). Predictability is the inverse of uncertainty, which is known to be aversive. For instance, there is evidence that the creation of response uncertainty by priming alternative incorrect responses triggers negative affective states to signal the need for conflict resolution to cognitive-control systems (van Steenbergen et al., 2009, 2015). Along the same lines, uncertainty in predicting the behavior of another person would result in the activation of multiple representations of possible actions, which induces a kind of response conflict. As this generates negative affective states, the negativity of the state would directly indicate the degree of appropriate trust. This need not be the only informational source that people consider when judging the trustworthiness of someone (e.g., social values may make one trust irrespective of one's feelings: Joosten et al., 2015), but we assume that it does make a particularly important contribution.

Considering the connection between predictability and trust, it makes sense to assume that trust increases as a function of the amount of knowledge available to predict the behavior of someone. Given that the most knowledge we have about ourselves (at least if it comes to the prediction of behavior), we would not only be able to predict the behavior of ourselves the best but also be good in predicting the behavior of people that are very similar to us. According to our theoretical approach, the representations of oneself and of another person differ only in degrees, which suggests that the degree of me-other similarity or congruency determines how much one trusts another. As this similarity/congruency depends on the amount of situationally relevant features that one shares with the other and the exclusivity/inclusivity of one's current processing mode, we can thus predict that the degree of trust between trustor and trustee should increase with the situationally relevant interpersonal similarity in the inclusivity of the trustor's processing mode.

## Improving trust

Our approach suggests basically three ways to improve (or reduce) interpersonal trust. First, if trust is derived from the degree of negative affect, it should be affected by emotional factors. While the degree of negative affect would normally reflect the degree of uncertainty-induced conflict, conflict-driven affective cues (that we assume to provide input for trust calculation) can be overwritten by external reward (van Steenbergen et al., 2009) and positive mood (van Steenbergen et al., 2010), suggesting that trust could be enhanced by these two factors. Indeed, Mislin et al. (2015) showed that trust increases as a function of both the trustor's mood and the received reward. These two factors interacted with one compensating for the other, suggesting that they indeed relate to the same mechanism. However, note that our approach over-justifies the impact of emotional factors in two ways. For one, positive mood and affect can not only overwrite conflict-induced negative affective signals but also induce more integrative processing modes (Dreisbach and Goschke, 2004)—which we also assume to increase trust. While this makes emotion-related predictions even stronger, it also makes successful predictions difficult to trace back to the actual mechanism. For another, mood and processing modes seem to share neural mechanisms and may thus not constitute separable causal factors: Relative increases in striatal dopamine seem to constitute central components of both positive mood (Akbari Chermahini and Hommel, 2012a) and more integrative control states (Cools, 2006), and not only does positive-going mood make processing modes more integrative but engaging in integrative processing also improves mood (Akbari Chermahini and Hommel, 2012b).

Second, if trust relates to self-other similarity, it should increase with this similarity. Indeed, facial similarity between trustor and trustee promotes attributions of trustworthiness (DeBruine, 2005; Bailenson et al., 2008) and cooperation in trust-related economic games (DeBruine, 2002; Krupp et al., 2008). Reversely, faces of people that either look (Verosky and Todorov, 2010) or behave (Farmer et al., 2013) untrustworthy are viewed as less similar to oneself.

Third, if trust is sensitive to the processing mode, it should be more pronounced under conditions that promote a more inclusive mode. To test this prediction, Sellaro et al. (2014a) have used creativity tasks to bias the processing mode of trustors before playing a Trust Game (Camerer and Weigelt, 1988). Previous studies have shown that engaging in convergent thinking promotes a focused, exclusive processing mode while engaging in divergent thinking promotes a distributed, inclusive processing mode (e.g., Fischer and Hommel, 2012). As predicted, trustors transferred significantly more money to trustees after engaging in divergent thinking as compared to convergent thinking, which confirms our prediction that an inclusive processing mode promotes trust.

Another study used the food supplement L-tryptophan, a precursor of serotonin, to promote interpersonal trust. Tryptophan depletion was found to improve performance in tasks that require focusing on relevant and ignoring irrelevant information, such as the Stroop task, in healthy and depressed participants (Rowley et al., 1998; Schmitt et al., 2000; Booij et al., 2005). This suggests that tryptophan depletion promotes a more focused, exclusive processing mode, which in turn implies that the administration of tryptophan promotes an integrative mode. Colzato et al. (2013a) exposed healthy participants to a dose of either tryptophan or a placebo before having them engage in a Trust Game. As predicted, trustors transferred more money to their trustees after the intake of tryptophan. Along those same lines, Steenbergen et al. (2014) reported that the administration of tryptophan increases the probability to engage in charitable donation.

Finally, there is evidence that trust can be improved by means of fragrance. Available evidence suggests that arousing fragrances, like peppermint, induce a more exclusive processing mode while calming scents, like lavender, induce a more integrative mode (e.g., Herz, 2009). Accordingly, Sellaro et al. (2014b) had participants play a Trust Game while being exposed to the scent of peppermint or lavender. As predicted, trustors transferred significantly more money to trustees in the lavender as compared to the peppermint and a control condition.

## Conclusion

We suggest that interpersonal trust is a function of the perceived similarity between trustor and trustee, and have suggested a concrete functional mechanism how this similarity is represented in the cognitive system. The available evidence provides support for three key predictions of our approach: that trust increases with positive emotions and the degree of functional trustor-trustee feature overlap, and with a more integrative processing mode of the trustor. Other implications of our approach were not yet tested, such as the assumption that perceived self-other similarity is mediated by the situational relevance of the represented features, which calls for studies in which this relevance is systematically varied. Nevertheless, we consider it interesting that social behavior can be predicted based on a model that was originally developed to account for entirely nonsocial agent-environment interactions. Also of interest, we have successfully applied the same approach to account for phenomena of social conformity (Kim and Hommel, 2015), which provides converging evidence that important kinds of social behavior may be generated by not dedicatedly social, domain-general cognitive mechanisms.

### Conflict of interest statement

The authors declare that the research was conducted in the absence of any commercial or financial relationships that could be construed as a potential conflict of interest.
